# A Pituitary Society update to acromegaly management guidelines

**DOI:** 10.1007/s11102-020-01091-7

**Published:** 2020-10-20

**Authors:** Maria Fleseriu, Beverly M. K. Biller, Pamela U. Freda, Monica R. Gadelha, Andrea Giustina, Laurence Katznelson, Mark E. Molitch, Susan L. Samson, Christian J. Strasburger, A. J. van der Lely, Shlomo Melmed

**Affiliations:** 1 Pituitary Center, Departments of Medicine and Neurological Surgery, Oregon Health & Science University, Portland, OR USA; 2 Neuroendocrine Unit, Massachusetts General Hospital, Harvard Medical School, Boston, MA USA; 3 Department of Medicine, Columbia University, Vagelos College of Physicians and Surgeons, New York, NY USA; 4 Neuroendocrinology Research Center/Endocrinology Section, Medical School and Hospital Universitário Clementino Fraga Filho, Universidade Federal Do Rio de Janeiro, Rio de Janeiro, Brazil; 5 Institute of Endocrine and Metabolic Sciences, San Raffaele Vita-Salute University and IRCCS San Raffaele Hospital, Milan, Italy; 6 Departments of Medicine and Neurosurgery, Stanford University School of Medicine, Stanford, CA USA; 7 Division of Endocrinology, Metabolism & Molecular Medicine, Northwestern University Feinberg School of Medicine, Chicago, IL USA; 8 Pituitary Center, Departments of Medicine and Neurosurgery, Baylor College of Medicine, Houson, TX USA; 9 Department of Medicine for Endocrinology, Diabetes and Nutritional Medicine, Charité Universitätsmedizin, Berlin, Germany; 10 Pituitary Center Rotterdam, Endocrinology Section, Department of Internal Medicine, Erasmus University Medical Center, Rotterdam, The Netherlands; 11 Pituitary Center, Cedars-Sinai Medical Center, 8700 Beverly Blvd., Room 2015, Los Angeles, CA 90048 USA

**Keywords:** Pituitary adenoma, Acromegaly, Growth hormone, Insulin-like growth factor I, Somatostatin receptor ligand, Pegvisomant, Oral octreotide

## Abstract

Guidelines and consensus statements ensure that physicians managing acromegaly patients have access to current information on evidence-based treatments to optimize outcomes. Given significant novel recent advances in understanding acromegaly natural history and individualized therapies, the Pituitary Society invited acromegaly experts to critically review the current literature in the context of Endocrine Society guidelines and Acromegaly Consensus Group statements. This update focuses on how recent key advances affect treatment decision-making and outcomes, and also highlights the likely role of recently FDA-approved therapies as well as novel combination therapies within the treatment armamentarium.

## Introduction

Guidelines and consensus statements ensure that physicians managing acromegaly patients have access to current information on evidence-based treatments to optimize outcomes.

Given significant novel recent advances in understanding acromegaly natural history and individualized therapies, the Pituitary Society invited acromegaly experts to critically review the current literature in the context of Endocrine Society guidelines [1] and Acromegaly Consensus Group statements [2, 3].

This update focuses on how recent key advances affect treatment decision-making and outcomes, and also highlights the likely role of recently FDA-approved therapies as well as novel combination therapies within the treatment armamentarium. Key summary points are presented in Tables 1, 2, 3. Grading of evidence and recommendations are described in Table 4.

**Table 1:** Presentation, monitoring, and outcomes: summary points

*Presentation, comorbidities, and mortality*
Although men present at a younger age than do women, women may show both increased incidence and mortality risk. (MQ, DR) Biochemical control remains the strongest predictor of patient outcomes, reflecting improvements in glucose metabolism, OSA, cardiovascular disease, and VFs. However, structural heart and joint changes are unlikely to resolve. (MQ, DR) The observed decline in reported mortality among acromegaly patients is likely due to more effective therapies, which, in turn, yield higher biochemical control rates and reduce the likelihood of developing respiratory and cardiovascular comorbidities that increase mortality. Rate of thyroid malignancies is not greater among acromegaly patients than among those without the condition. After screening colonoscopy at diagnosis, further testing should be performed similar to the general population, as per previous recommendations. (LQ, DR)
*Assays*
Reference GH nadir levels after OGTT using the IDS-iSYS assay accounting for BMI, sex, and ethinylestradiol-containing oral contraceptive use confirm the importance of these factors as confounders in GH measurements. (MQ, SR) IGF-I levels measured 6 weeks postoperatively can be used in most patients to assess remission, although patients with mildly elevated IGF-I may yet normalize by 3–6 months. (MQ, SR)
*Sex, age, and surgical outcomes*
Women, especially when postmenopausal, may exhibit lower surgical remission rates from TSS, as they tend to have larger and more invasive tumors that are less amenable to total resection. (LQ, DR) Patient age is likely not a predictor of surgical outcomes, nor does it impact the favorable effects of postsurgical remission on alleviating disease comorbidities. (LQ, DR)
*Radiotherapy outcomes*
Long-term follow-up of patients treated with SRS and FRT show that approximately half achieve and maintain biochemical control. However, up to one-third of patients with normal pituitary function develop hypopituitarism, confirming the need for ongoing monitoring. (LQ, SR)

*BMI* body mass index; *DR* discretionary recommendation; *FRT* fractionated radiotherapy; *GH* growth hormone; *IGF-I* insulin-like growth factor I; *LQ* low-quality evidence; *MQ* medium-quality evidence; *OGTT* oral glucose tolerance test; *OSA* obstructive sleep apnea; *SR* strong recommendation; *SRS* stereotactic radiosurgery; *TSS* transsphenoidal surgery; *VF* vertebral fracture

**Table 2:** Medical therapy: summary points

*Injectable SRL*
Older age, female sex, lower IGF-I levels, and tumor T2 MRI hypointensity at baseline predict more favorable long-term biochemical responses to primary lanreotide 120 mg therapy every 4 weeks. (MQ, SR) Recent studies confirm that extended-dosing intervals (> 4 weeks) for 120 mg lanreotide may be effective among selected patients previously controlled with long-acting SRLs. (LQ, DR) Several studies confirm efficacy of pasireotide LAR for some patients uncontrolled on lanreotide or octreotide LAR. However, rates of treatment-induced hyperglycemia and DM are high, requiring careful monitoring for glycemic side effects. (HQ, SR)
*Pegvisomant*
Ten-year follow-up from ACROSTUDY shows a 73% biochemical control rate with very low rates of transient elevated transaminases and 6.8% exhibiting tumor growth visible on MRI. (HQ, SR) Pegvisomant use in patients with DM improves glucose metabolism independent of IGF-I control, but does not affect glycemic endpoints in patients without DM. (MQ, SR) Patients with DM and those with a higher BMI require higher doses of pegvisomant and more rapid up-titration to achieve IGF-I normalization. (MQ, SR)
*Combination therapy with SRL* + *pegvisomant*
Low-dose octreotide LAR or lanreotide plus weekly pegvisomant is a cost-effective and efficacious option for patients requiring combination therapy. (HQ, SR) Combination of pasireotide plus pegvisomant can yield biochemical control rates exceeding 70% even when pegvisomant doses are kept low. However, the addition of pegvisomant does not ameliorate the high rates of pasireotide-induced hyperglycemia. (MQ, SR) Patient selection for combination pasireotide plus pegvisomant should be carefully considered. (LQ, DR)

*BMI* body mass index; *DM* diabetes mellitus; *DR* discretionary recommendation; *HQ* high-quality evidence; *IGF-I* insulin-like growth factor I; *LAR* long-acting release; *LQ* low-quality evidence; *MQ* medium-quality evidence; *MRI* magnetic resonance imaging; *SRL* somatostatin receptor ligand

**Table 3:** Oral octreotide capsules: recommendations

*How should OOC be integrated into the current treatment algorithm for medical management of acromegaly?*
OOC are suitable for patients who have demonstrated complete or partial biochemical response on injectable octreotide or lanreotide. (HQ, SR) *Rationale:* As octreotide and lanreotide have similar efficacy, patients who have responded to these injectable agents are candidates for OOC therapy, and results of the OPTIMAL study demonstrate that biochemically controlled patients (IGF-I ≤ 1.0 × ULN) on stable doses of injectable octreotide or lanreotide maintain response to OOC [4]. There are no data regarding efficacy of switching patients from pasireotide LAR to OOC.There are no data on the use of OOC as primary medical therapy in SRL-naïve patients. However, it is reasonable to expect that patients who respond to injectable octreotide LAR or lanreotide in this setting would also respond to OOC
Due to a lack of available data, OOC is not currently recommended for patients who have tumor characteristics predictive of octreotide resistance. (MQ, SR) *Rationale:* Tumor characteristics associated with octreotide and lanreotide resistance (e.g., MRI T2 hyperintensity, sparsely granulated tumors) [5, 6] are presumed to also predict resistance to OOC
*How should OOC be initiated?*
OOC is initiated at a dose of 40 mg/day, given as 20 mg capsules twice per day taken 1 h before a meal or 2 h after a meal to maximize bioavailability. (MQ, SR) However, clinical study data suggest a starting dose of 60 mg/d may be optimal for most patients. *Rationale:* The 40 mg/day dose is the approved initiation dose [7]. Most responders in the OPTIMAL study up-titrated to 60 mg/d or 80 mg/d by study end, and all patients enrolling in the open label extension study were reinitiated at the 60 mg/d dose [4, 8]
OOC should be initiated at the time of the previously scheduled SRL injection. (HQ, SR) *Rationale:* In clinical trials, OOC was initiated at the time of the next SRL injection, i.e., at the end of the once-monthly injection period [4, 9]. IGF-I levels may increase toward the end of the injection period with waning of injectable drug levels [10], and likely account for reported exacerbation of acromegaly symptoms [11–13]
*How should OOC dose be escalated?*
OOC can be up-titrated by an increment of 20 mg every 2–4 weeks based on IGF-I and clinical symptoms. (MQ, SR) *Rationale:* The pharmacokinetics of OOC [14] enable a dose titration every 2–4 weeks. This is a more rapid escalation compared with injectable SRLs, which often are up-titrated every 3 months. Slower titration may risk re-emergence of disease signs and symptoms and loss of biochemical control

*HQ* high-quality evidence; *IGF-I* insulin-like growth factor I; *MQ* medium-quality evidence; *MRI* magnetic resonance imaging; *OOC* oral octreotide capsules; *SR* strong recommendation; *SRL* somatostatin receptor ligand; *ULN* upper limit of normal

**Table 4:** Evidence and recommendations grading

*Evidence*	
Very low quality (VLQ)	Expert opinion supported by one or few small uncontrolled studies
Low quality (LQ)	Supported by large series of small uncontrolled studies
Moderate quality (MQ)	Supported by one or few large uncontrolled studies or meta-analyses
High quality (HQ)	Supported by controlled studies or large series of large uncontrolled studies with sufficiently long follow-up
*Recommendations*	
Discretionary recommendation (DR)	Based on VLQ or LQ evidence
Strong recommendation (SR)	Based on MQ or HQ evidence

Adapted from Giustina et al. [2]

## Presentation, comorbidities, and mortality

### What’s new

A better understanding of acromegaly natural history is emerging from recent studies. A population-based case–control study from Korea including 718 patients showed that acromegaly incidence is slightly higher in females [15], consistent with some, but not all other earlier studies [16, 17].

Yet, nearly all studies concur that men are significantly younger than women at diagnosis, by a median of 4.5 years [18].

Risks of complications and comorbidities associated with acromegaly are lower in patients who are biochemically controlled [19]. Of note, older age confers the same increased risk for diabetes mellitus (DM), hypertension, sleep apnea, and cancer as in the general population [20], while left ventricular hypertrophy is more frequent among elderly patients with acromegaly [21].

A retrospective study of 150 patients treated at a single center for a median of 10.4 years [22] assessed treatment and disease control impact on acromegaly comorbidities. Biochemical control, assessed only by a random growth hormone (GH) level < 2.5 μg/L, was associated with a lower hazard ratio (HR) of developing DM (HR 0.36; 95% CI 0.15, 0.83; p = 0.017) as well as cardiovascular system disorders overall (HR 0.54; 95% CI 0.31, 0.93; p = 0.027) compared to those not controlled. However, the risks of developing arterial hypertension and myocardial hypertrophy were not different [22]. An increased risk of arthropathy was also noted (HR 1.68; 95% CI 1.04, 2.71; p = 0.032), suggesting that, once established, structural changes are less likely to be influenced by biochemical control.

Importantly, acromegaly treatment improves glucose metabolism even if IGF-I is not normalized [23]. Surgical tumor remission, although achieved in only 41% of 64 treatment-naïve patients in one study, resulted in reduced DM rate, from 28% before surgery to 8% after, while normal glucose tolerance increased from 29% to 62.5% [23].

Biochemically active disease is generally associated with a higher risk of vertebral fractures (VF) [19, 24]. One study [25] involved 55 patients treated with pasireotide long acting release (LAR) or pegvisomant who had been previously uncontrolled on octreotide LAR or lanreotide for at least 6 months, 42% of whom had VFs at baseline. After a median of 36 months follow-up, 67% of patients treated with pasireotide LAR and 77% treated with pegvisomant achieved disease control. Intriguingly, among those with active disease, incident VFs were significantly less frequent among those treated with pasireotide than with pegvisomant (78% vs 25%, p = 0.04), regardless of IGF-I level during follow-up. The mechanisms underlying this finding are unclear, but may include differential impact of pegvisomant vs pasireotide on GH signaling in bone or an independent effect of somatostatin receptor ligands (SRL) on bone turnover.

No controlled studies on bone active agents in the prevention and treatment of vertebral fractures are available. A multicenter observational study of 111 patients with active acromegaly [26] suggested that, in general, use of of bone active drugs may be associated with lower risk of incident VFs (OR 0.11; p = 0.004). As patients were treated with a wide variety of agents, these findings cannot be applied to use of any one specific agents. Selective estrogen receptor modulators may prove a particularly interesting option due their potential dual effect on both bone health [27] and acromegaly control [28]. However, patients with controlled acromegaly can continue to develop VFs. In a 9.1-year prospective follow-up study [29], VFs progressed in 11/31 (35.5%), with patients post-surgery or post-radiation demonstrating a higher risk of VF progression (p = 0.030).

Improved biochemical control is also associated with a reduction in obstructive sleep apnea (OSA) and apnea–hypopnea index (AHI) assessed by polysomnography. A meta-analysis [30] that included 24 studies (n = 734) showed significant AHI improvement after medical or surgical treatment (effect size − 0.36; 95% CI − 0.49, − 0.23; p < 0.001), and another study of 27 patients [31] showed that 69% of patients with OSA at baseline were cured after achieving acromegaly disease control.

Other studies confirmed beneficial effects of acromegaly treatment on health-related quality of life (QOL). QOL improved but did not normalize in a prospective study of 27 patients followed for 2.5 years after diagnosis, all of whom achieved disease control with surgery and/or medical therapy, especially in the first year of treatment [32]. Results of the longitudinal surveillance ACROSTUDY similarly showed improvement in QOL with pegvisomant using both AcroQoL and PASQ questionnaires [33]. A patient-centric approach for QOL assessment may allow a more personalized method of management [34], and tools such as the recently developed Acromegaly Treatment Satisfaction Questionnaire (Acro-TSQ) also show improved QOL with disease control [11], specifically in patients treated with injectable SRL [35]. However, addressing the high discordance between patient- and medical provider-reported symptom severity, pattern of acromegaly symptoms, and treatment injection site reactions remains a challenge for treating physicians [12]. The relationship between sex and QOL remains unclear due to heterogeneous methods and design, assessment tools, and patient cohorts [36, 37].

Over the past decade, disease control has improved due to enhanced therapeutic strategies, leading to reversal of the increased mortality risk traditionally associated with acromegaly [38–40]. A meta-analysis showed increased mortality in 17 studies published before 2008 (standardized mortality ratio [SMR] 1.76; 95% CI 1.52, 2.4; p < 0.00001), but mortality was strikingly not different from the general population in 9 studies published after 2008 (SMR 1.35; 95% CI 0.99, 1.85) [39]. Similar results were reported in a retrospective study in Sweden of 1089 patients with acromegaly analyzed for three periods (1987–1995, 1996–2004, and 2005–2013) based on the year of diagnosis [40]. SMR for the group overall was 2.79 (95% CI 2.43, 3.15) compared with the general population, but mortality decreased over time, with an SMR of 3.45 (95% CI 2.87, 4.02) and 1.86 (95% CI 1.04, 2.67) during the first and last time period, respectively (p = 0.015). Although mortality in patients with controlled acromegaly is generally similar between males and females as in the overall population [19], the recent nationwide Korean study [15] found that females, but not males, with acromegaly showed a higher mortality risk compared with age- and sex-matched controls (HR 1.75; 95% CI 1.07, 2.84).

Excess mortality reported in earlier studies was primarily due to cardiovascular diseases (SMR 2.95; 95% CI 2.35, 3.55), including ischemic heart disease (SMR 2.00; 95% CI 1.35, 2.66) and cerebrovascular disease (SMR 3.99; 95% CI 2.42, 5.55), with a lesser effect from malignancy (SMR 1.76; 95% CI 1.27, 2.26) [40]. In recent studies, cancer has been reported as the leading cause of death in acromegaly, likely related to longer life expectancy due to better control of the disease and its related comorbidities rather than a specific increased risk of cancer [38, 39, 41]. A nationwide cohort from Taiwan including 1195 patients followed from 1997 to 2013 showed 87 newly diagnosed cancers, with an incidence rate of 10.6 per 1,000 person-years [42], or a standardized incidence ratio of 1.91. However, studies of the two cancers most associated with acromegaly, namely colon and thyroid cancer [19, 43], suggest that this risk might not be clinically significant. Comparing 178 patients and 356 controls, colorectal polyps were found in 67% of patients in the acromegaly group and in 24% of the control group (p < 0.001), but there was no difference in histology subtypes [44].

### Summary points

- Although men present at a younger age than do women, women may show both increased incidence and mortality risk.
- Biochemical control remains the strongest predictor of patient outcomes, reflecting improvements in glucose metabolism, OSA, cardiovascular disease, and VFs. However, structural heart and joint changes are unlikely to resolve.
- The observed decline in reported mortality among acromegaly patients is likely due to more effective therapies, which, in turn, yield higher biochemical control rates and reduce the likelihood of developing respiratory and cardiovascular comorbidities that increase mortality. The rate of thyroid malignancies is not greater among acromegaly patients than among those without the condition. After screening colonoscopy at diagnosis, further testing should be performed similar to the general population, as per previous recommendations.

## Assays

### What’s new

As GH and IGF-I assessments remain the standard for measuring acromegaly disease activity at diagnosis and follow-up, strategies are being developed to improve current assays. Reference nadir levels of GH using the IDS-iSYS GH assay during oral glucose tolerance testing (OGTT) that account for body mass index (BMI), sex, and estradiol-containing oral contraceptives (OC) have been empirically established [45]. Dividing 525 non-acromegalic individuals into cohorts with BMI < 25 vs ≥ 25 kg/m^2^, the leaner group had GH nadirs more than twice as high as the heavier cohort (0.22 vs 0.09 µg/L, p < 0.0001), while pre- but not postmenopausal women had higher GH nadir vs men and mean GH nadir in OC-using females exceeded by more than threefold the GH nadir mean of premenopausal women not using OC [45].

Other markers of GH action such as IGF binding protein 3 or acid-labile subunit have been suggested to assess discrepant GH and IGF-I results [46]. Soluble Klotho, predominantly expressed in the kidney [47], correlates with GH levels over a wide concentration range [48], and has been suggested to correlate with QOL improvements [49].

Defining postoperative remission using IGF-I is a well-recognized challenge, as it may require 3 months to achieve a steady plateau [50]. Retrospective data on 69 patients [51] suggest that IGF-I measured 6 weeks postoperatively may be an early indicator of disease activity in most patients, but repeat assessment is warranted at 3–6 months for those with IGF-I levels mildly elevated above the age-related normal range, no cavernous sinus invasion, and postoperative GH < 1 ng/mL, as IGF-I may yet normalize.

### Summary points

- Reference GH nadir levels after OGTT using the IDS-iSYS assay accounting for BMI, sex, and estradiol-containing oral contraceptive use confirm the importance of these factors as confounders in GH measurements.
- IGF-I levels measured 6 weeks postoperatively can be used in most patients to assess remission, although patients with mildly elevated IGF-I may yet normalize by 3–6 months.

## Sex, age, and surgical outcomes

### What’s new

Recent studies suggest that female sex, but not age, may impact surgical outcomes. A large retrospective single-center study of 463 patients who underwent transsphenoidal surgery (TSS) found that women had lower pre-operative IGF-I compared with men, yet were older at surgery and had larger adenomas and more cavernous sinus invasion. Accordingly, rates of total tumor resection were significantly higher in men than in women (92.6% vs 85.5%; p = 0.021), as were rates of remission postsurgery (89.7% vs 76.5%; p < 0.001) [52]. Another single-center retrospective study similarly showed that women had larger tumors despite lower mean IGF-I levels, although there were no differences in histological granulation patterns [53]. Of note, premenopausal women tended to have larger, more aggressive tumor types and lower remission rates than men [52], suggesting a more aggressive natural history and hence more adverse treatment outcomes in this subset of women. Models that yield much higher predictive values than each individual parameter are being developed [54].

Data on acromegaly in the elderly are sparse [20]. One tertiary care center study of 57 patients age ≥ 65 years reported a surgical remission of 73.7% [55]. These patients tended to have smaller adenomas with lower invasion rates, which may explain why other studies [56] did not show age as a predictor of remission. Of note, a study of 87 consecutive patients who underwent TSS showed no significant between-group differences in perioperative complications and/or endocrinological remission comparing those younger and older than 65 years. Incidence of new postoperative pituitary deficiency was also similar, and remission enabled one-third of patients over age 65 years to stop medication for hypertension and DM [57].

### Summary points

- Women, especially when postmenopausal, may exhibit lower surgical remission rates from TSS, as they tend to have larger and more invasive tumors that are less amenable to total resection.
- Patient age is likely not a predictor of surgical outcomes, nor does it impact the favorable effects of postsurgical remission on alleviating disease comorbidities.

## Injectable SRL

### What’s new

Identifying populations most likely to benefit from long-acting injectable SRLs is important. In the PRIMARYS study of lanreotide 120 mg in patients with treatment-naive macroadenomas, ≥ 20% tumor volume reduction was achieved in 54% at 12 weeks and in 63% at 48 weeks or the last post-baseline visit available [58]. Older age, female sex, and lower IGF-I levels at baseline were associated with increased probability of achieving long-term biochemical control, but tumor volume response at 12 weeks was not an accurate predictor of subsequent tumor volume control [59]. Further, patients with a hypointense tumor on T2 MRI showed greater reductions in IGF-I and were more likely to achieve tumor shrinkage [60]. These results suggest that patient- and tumor-specific factors at baseline may predict long-term biochemical response to primary SRL treatment, while early tumor response may not. Moreover, meta-analysis of 622 patients from two European cohorts using multivariable regression models found that baseline IGF-1 was the best predictor of biochemical response to octreotide and lanreotide, followed by body weight; younger patients were more likely to be nonresponsive [61].

In a prospective international study, 88.7% of patients well controlled on octreotide LAR 10 and 20 mg every 4 weeks who switched to lanreotide 120 mg every 6 weeks achieved normal IGF-I levels after 24 weeks [62]. Such extended-dosing intervals may be effective in patients who have achieved good biochemical control with long-acting SRLs [3].

The phase 3 PAOLA study, which randomized 198 acromegaly patients uncontrolled on octreotide LAR or lanreotide to continued treatment or pasireotide, found that 15% and 20% of patients treated with pasireotide 40 mg and 60 mg, respectively, achieved biochemical control after 24 weeks vs 0% in the octreotide/lanreotide group [63]. Using a cutoff of GH < 1.0 μg/L and normal IGF-I to define disease control in the extension study, after a mean follow-up of 304 weeks (5.8 years) for the 111 patients initially randomized to pasireotide and 268 weeks (5.2 years) for the 62 patients in the crossover group, 37% achieved control at some point during the study, and 65.5% of these achieved a first response after at least 6 months of treatment. Escalating pasireotide doses from 40 to 60 mg allowed 28% to achieve disease control, while switching to pasireotide from octreotide/lanreotide enabled control in 22% of patients [64].

An open-label study similarly switched uncontrolled patients from octreotide/lanreotide to pasireotide, but used a more rigorous cutoff for biochemical control (GH < 1.0 μg/L and normal age-matched IGF-I levels) for both the 36-week core phase and an additional 36-week extension phase [65]. Among 123 patients treated in the core phase, 15% achieved normal GH and IGF-I and 31% achieved only normal IGF-I after switching to pasireotide, with higher rates among those with GH 1.0–2.5 μg/L at baseline [65]. At baseline, 42% were diabetic and 49% pre-diabetic; during the study, 42% reported new-onset hyperglycemia and 24% DM.

The greater risk of drug-induced hyperglycemia and DM with pasireotide likely results from impaired insulin and incretin secretion, with a minor effect on glucagon production [66]. Prevalence of DM in PAOLA was 26% [63], and post-hoc analysis showed that patients with impaired fasting blood glucose (FBG; > 100 mg/dL) at baseline were more likely to develop glycometabolic abnormalities [67]. In the extension study, hyperglycemia was reported in 40% of patients who continued on pasireotide and in 26% of those who crossed over from octreotide/lanreotide, while DM was reported in 32% of patients treated with 40 mg pasireotide, 40% of those treated with 60 mg pasireotide, and 29% of those who crossed over from octreotide/lanreotide [64]. Generally, the degree of hyperglycemia associated with pasireotide is largely dependent on glycemic control at baseline [65, 68]. Importantly, most patients are successfully managed with concomitant antidiabetic medications and show a glycated hemoglobin (HbA1c) level < 7% [69]; few patients discontinue treatment due to hyperglycemia [65, 69].

Smaller observational studies of previously uncontrolled patients who switched to pasireotide monotherapy showed a higher (54%) rate of IGF-I normalization [70] or similar efficacy compared with combination octreotide/lanreotide plus pegvisomant [71]. However, hyperglycemia and DM were prevalent, with 17/22 patients in one study requiring initiation or intensification of antidiabetic medication [70] and 6/15 in another requiring antidiabetic therapy as early as 15 days after initiating treatment [71].

Results of these studies support current recommendations for pasireotide use, which note the need for careful screening and monitoring of glycemic side effects and a preference for use in octreotide/lanreotide-refractory patients with normal glucose metabolism [3].

### Summary points

- Older age, female sex, lower IGF-I levels, and tumor T2 MRI hypointensity at baseline predict more favorable long-term biochemical responses to primary lanreotide 120 mg therapy every 4 weeks.
- Recent studies confirm that extended-dosing intervals (> 4 weeks) for 120 mg lanreotide may be effective among selected patients previously controlled with long-acting SRLs.
- Several studies confirm efficacy of pasireotide LAR for some patients uncontrolled on lanreotide or octreotide LAR. However, rates of treatment-induced hyperglycemia and DM are high, requiring careful monitoring for glycemic side effects.

## Oral octreotide capsules

### What’s new

Oral octreotide capsules (OOC) received regulatory approval from the US Food and Drug Administration in June 2020 for long-term maintenance treatment in acromegaly patients who have responded to and tolerated treatment with octreotide or lanreotide.

OOC contains unmodified octreotide suspended within a lipophilic medium of the medium-chain fatty acid sodium caprylate within an enteric coated gelatin capsule. Released octreotide is absorbed by a paracellular route, via transient openings in tight junctions between intestinal epithelial cells [14, 72]. Studies in healthy volunteers established that 20 mg OOC has similar pharmacokinetics to subcutaneous injection of 0.1 mg of subcutaneous (SC) octreotide, with comparable half-lives of 2.25 and 2.38 h respectively [14]. However, after a standardized meal, octreotide from OOC lost 90% of bioavailability. Thus, careful administration of the capsules timed to meals is essential.

In a phase 3 open-label, multicenter trial [9], 155 patients controlled on injectable SRLs for ≥ 3 months (IGF-1 ≤ 1.3 × ULN) were switched to OOC in two daily divided doses starting with 40 mg/day (20 mg BID) and titrating up to 60 mg/day (40 mg + 20 mg) or 80 mg/day (40 mg BID) at least 1 h before or more than 2 h after a meal. The dose-escalation period of 2–5 months was followed by a 7-month fixed-dose core period and a voluntary 6-month extension phase. The primary endpoint of IGF-I < 1.3 × ULN and integrated GH < 2.5 ng/L was achieved by 65% of 151 evaluable participants at the end of the core phase and 62% at the end of the extension phase, with 85% of controlled patients maintaining biochemical response. Two hours after the first dose, mean integrated GH levels markedly decreased from 0.77 ng/mL at baseline to 0.40 ng/mL, which was maintained through to the end of the study (mean, 0.49 ng/mL) [9].

The phase 3 randomized, placebo controlled, double blinded OPTIMAL trial [4] used more stringent entry criteria of mean IGF-I ≤ 1 × ULN with the same dosing schema as in the open-label phase 3 study (40 mg/day up to 80 mg/day). Patients could be reverted to their previous injectable SRL if IGF-I was ≥ 1.3 × ULN for 2 consecutive visits while on the highest dose of OOC (80 mg/day) or placebo accompanied by worsening clinical signs or symptoms of acromegaly. A total of 56 patients were enrolled and randomized 1:1 to OOC treatment or placebo for 36 weeks. The primary endpoint of IGF-I ≤ 1 × ULN was achieved by 58% of patients in the OOC group compared to 19% on placebo (p = 0.008). This analysis imputed non-response for missing data (i.e., worst observation carried forward). Applying the more commonly used last observation carried forward imputation, the response rate was 64.3%. The authors noted that the higher than expected placebo response rate is most likely due to IGF-I variability throughout the study, including loss of response for two consecutive visits [4]. Significant differences were seen with OOC vs placebo for the proportion of patients who maintained GH response at 36 weeks, time to loss of response, and proportion of patients who began reversion to prior treatment prior to and including week 36. By study end, most patients responding to OOC (11/16; 68.8%) had been up-titrated to 60 mg/day or 80 mg/day [73]. The open-label extension (OLE) study reinitiated all patients at a starting dose of 60 mg OOC [8]; of the 40 patients enrolled, 3 required dose decrease to 40 mg and 27 patients required increased dose to 80 mg, with 93% maintaining response.

Adverse events (AEs) were as expected for octreotide. There were no treatment-related serious AEs and no dose-related AE patterns. Almost all patients on placebo and half on OOC reported signs and symptoms that could be attributable to acromegaly. Among the 25% of patients treated with OOC who required reversion to injectable SRL due to treatment failure or AEs, IGF-I levels returned to baseline within a median of 4 weeks and baseline response was re-established after a single SRL injection [4]. Interestingly, despite the higher starting dose, overall incidence of AEs was lower in the OLE than in the main study (57.9% vs 96.4%), further demonstrating that AEs are not dose related and supporting use of a higher starting dose of 60 mg/day.

### Recommendations

Injectable octreotide LAR is a well-established treatment for acromegaly [74], and guidance is needed for how a daily oral formulation of octreotide should best be used in practice. Rationale for each recommendation is given in Table 3.

- OOC are suitable for patients who have demonstrated complete or partial biochemical response on injectable octreotide or lanreotide.
- Due to a lack of available data, OOC is not currently recommended for patients who have tumor characteristics predictive of octreotide resistance.
- OOC is initiated at a dose of 40 mg/day, given as 20 mg capsules twice per day taken 1 h before a meal or 2 h after a meal to maximize bioavailability. However, clinical study results suggest a starting dose of 60 mg/day may be the optimal starting dose for most patients.
- OOC should be initiated at the time of the previously scheduled SRL injection.
- OOC can be up-titrated by an increment of 20 mg every 2–4 weeks based on IGF-I and clinical symptoms. This is a more rapid escalation than is used with injectable SRLs, which often are up-titrated every 3 months.

## Pegvisomant

### What’s new

ACROSTUDY, the international, longitudinal surveillance study of patients treated with pegvisomant, continues to yield data important for optimizing pegvisomant use in clinical practice. At 10 years of follow-up, 73% of 2,090 patients had normal IGF-I levels [75]. Furthermore, mortality was the same as in the general population for patients with normalized IGF-I during treatment after a median of 4.1 years follow-up [76]. The updated analyses also confirm no new safety signals with long-term pegvisomant use. Most patients (72%) had no change visible on MRI, with 6.8% showing increased tumor size compared to prior scans [75]. In patients with normal liver function tests (LFTs) at baseline, 3% reported at least one transaminase elevation > 3 × ULN, and there were no cases of liver failure. Most elevations were transient and < 1% withdrew because of abnormal LFTs [75].

Not surprisingly, a systematic review and meta-analysis of observational longitudinal studies of pegvisomant showed similar results, as most of the included reports were based on ACROSTUDY data [77]. Overall, IGF-I control was observed in 71.7% of patients on pegvisomant monotherapy, with tumor growth noted in 7.3% and transaminase elevation in 3.0%. Real-word experiences independent of ACROSTUDY also mirrored these results, despite some country-specific differences in how pegvisomant is used in monotherapy vs combination therapy regimens [78–80]°

In 1,762 patients in ACROSTUDY, 29% of whom had DM at baseline (HbA1c ≥ 6.5%, FBG > 200 g/dL, or use of antidiabetic medication) [81], cross-sectional analysis at 4 years of follow-up showed that FBG and HbA1c remained stable in patients without DM, but prevalence of impaired glucose tolerance decreased from 11 to 8% at year 1 and 6.4% at year 4. Longitudinal analysis showed 53% of patients with DM and elevated IGF-I at baseline achieved IGF-I normalization by year 4, but decrease in IGF-I and glycemic change were not correlated.

Similar results were seen in a subset of 110 patients naïve or semi-naïve to pegvisomant (i.e., off drug for at least 6 months): median HbA1c improved from 5.8% to 5.6% at year 2 in patients with controlled acromegaly, but worsened from 6.1% to 6.3% in those with uncontrolled disease [33].

Meta-analysis of 13 prospective studies comprising 435 patients found that, independent of changes in IGF-I, fasting plasma glucose, fasting plasma insulin, HbA1c, and homeostatic model of assessment of insulin resistance (HOMA-I) all significantly improved with pegvisomant monotherapy, while only fasting plasma insulin improved in patients treated in combination with SRLs [82].

Of note, a higher mean dose of pegvisomant was needed in patients with DM (18.2 vs 15.3 mg/day), who also had a higher mean BMI compared with non-diabetic patients [81]. Results of a multicenter study of 87 patients [83] similarly showed that obese patients required a higher pegvisomant dose and a more rapid up-titration to achieve biochemical control. These results support an earlier ACROSTUDY report that found that the 56 patients who needed > 30 mg/day pegvisomant to achieve IGF-I normalization were younger, had higher BMI, and were also more likely to have DM, OSA, and hypertension [84]. These results suggest that dose up-titration may be needed in patients with DM and/or obesity to achieve normal IGF-I.

### Summary points

- Ten-year follow-up from ACROSTUDY shows a 73% biochemical control rate with very low rates of transient elevated transaminases and 6.8% exhibiting tumor growth visible on MRI.
- Pegvisomant use in patients with DM improves glucose metabolism independent of IGF-I control, but does not affect glycemic endpoints in patients without DM.
- Patients with DM and those with a higher BMI require higher doses of pegvisomant and more rapid up-titration to achieve IGF-I normalization.

## Combination therapy with SRL + pegvisomant

### What’s new

Combination therapy with pegvisomant plus SRL is increasingly being used in real-world settings [85]. In a single-center prospective study of 51 patients [86], a novel combination of low-dose monthly octreotide LAR (10 mg) or lanreotide (60 mg) combined with weekly pegvisomant (40 mg-160 mg/week) achieved a biochemical control rate of 96% in controlled and uncontrolled patients at considerably lower cost compared with combination regimens of higher-dose SRL and weekly pegvisomant or low-dose SRL and daily pegvisomant. Only 30% required up-titration of pegvisomant.

The PAPE study [87, 88] included 61 patients well-controlled on octreotide/lanreotide plus pegvisomant switched to pasireotide with or without pegvisomant. Following a 12-week run-in phase in which pegvisomant dose was reduced by 50%, 15 (25%) biochemically controlled patients were switched to 60 mg pasireotide monotherapy, while 46 (75%) uncontrolled patients were switched to the same dose of pasireotide but continued the 50% reduced pegvisomant dose (mean, 61 mg/week). At 24 weeks, or 12 weeks after switching, IGF-I was normalized in 73.8% of patients, including 93% of patients in the monotherapy arm and 67% of patients in the combination arm, despite decreasing mean pegvisomant to 48 mg/week and pegvisomant discontinuation in 68% of patients.

However, the rate of hyperglycemia was high, with significant increases between weeks 12 and 24 in mean fasting plasma glucose (6.1 to 9.1 mmol/L; p < 0.0001) and mean HbA1c (6.1% to 7.3%; p < 0.0001). New-onset DM was reported in 36.1%, doubling the prevalence of DM from 32.8% at baseline to 68.9% at 24 weeks. Although only 25% were receiving antidiabetic medication at baseline, after 24 weeks, 69% required at least one antidiabetic medication, most commonly metformin and/or a dipeptidyl peptidase 4 inhibitor. Of note, HbA1c levels were similar at both 24 and 48 weeks among those on pasireotide monotherapy and pasireotide plus pegvisomant combination therapy [87], indicating that improved glycemia seen with pegvisomant likely due to increased insulin sensitivity [81] does not ameliorate suppression of insulin secretion driving pasireotide-induced hyperglycemia [67]. Careful patient selection for this combination is recommended.

Additional analyses of PAPE data focused on predictors of treatment benefit. Neither GH nor IGF-I correlated with improved QOL observed after switching combination therapy regimens [49]. Somatostatin receptor SST2, but not SST5, expression, correlated with lower IGF-I levels [89]. Separate analysis of 13 tissue samples found that those with ≥ 25% decrease in tumor size had lower SST2 expression as well as lower SST2/SST5 ratio expression [90].

### Summary points

- Low-dose octreotide LAR or lanreotide plus weekly pegvisomant is a cost-effective and efficacious option for patients requiring combination therapy.
- Combination of pasireotide plus pegvisomant can yield biochemical control rates exceeding 70% even when pegvisomant doses are kept low. However, the addition of pegvisomant does not ameliorate the high rates of pasireotide-induced hyperglycemia.
- Patient selection for combination pasireotide plus pegvisomant should be carefully considered.

## Radiotherapy

### What’s new

In a single-center retrospective study of patients treated with single-fraction Gamma Knife stereotactic radiosurgery (SRS) between 1990 and 2017 [91] and followed for a median 63 months, 58 of 102 patients (57%) achieved biochemical control at a median of 19 months, and 22 patients persisted with active disease despite adjuvant medical treatment. Similar rates were seen in an update from the German Acromegaly Registry, which analyzed outcomes from both fractionated radiotherapy (FRT; n = 233) and SRS (n = 119) followed for up to 45 years [92]. Median time to achieve disease control was 3.0 years for FRT and 2.1 years for SRS, and the 10-year remission rate was 48% and 52% for FRT and SRS, respectively. Twenty-nine percent of patients developed hypopituitarism at a median of 29.5 months with SRS [91], while adrenocorticotropin (ACTH) and thyrotropin (TSH) deficiencies were more common with FRT than with SRS [92]. It should be noted that in all studies reporting radiotherapy outcomes, the patients were only a subset of those treated for acromegaly and were often those less responsive to prior surgery and medical treatments.

### Summary points

- Long-term follow-up of patients treated with SRS and FRT show that approximately half achieve and maintain biochemical control. However, up to one-third of patients with normal pituitary function develop hypopituitarism, confirming the need for ongoing monitoring.
